# Low Level Antibodies Against Alpha-Tropomyosin Are Associated With Increased Risk of Coronary Heart Disease

**DOI:** 10.3389/fphar.2020.00195

**Published:** 2020-02-27

**Authors:** Yin Zhang, Heru Zhao, Bin Liu, Li Li, Lulu Zhang, Mei Bao, Xinyu Ji, Xiaojuan He, Jianfeng Yi, Peng Chen, Cheng Lu, Aiping Lu

**Affiliations:** ^1^Key Laboratory for Research on Active Ingredients in Natural Medicine of Jiangxi Province, Yichun University, Yichun, China; ^2^Institute of Basic Research in Clinical Medicine, China Academy of Chinese Medical Sciences, Beijing, China; ^3^Beijing Key Laboratory of Traditional Chinese Medicine Basic Research on Prevention and Treatment for Major Diseases, Experimental Research Center, China Academy of Chinese Medical Sciences, Beijing, China; ^4^School of Chinese Medicine, Hong Kong Baptist University, Hong Kong, China

**Keywords:** coronary heart disease, autoantigens, autoantibodies, risk factors, protein microarrays

## Abstract

**Objective:**

Natural autoantibodies have been implicated to play a key role in the pathogenesis of coronary heart disease (CHD) because they augment autoimmune activation. The aim of this study was to identify novel specific autoantibodies of CHD, and analyze the relationship between their levels and CHD risk indicators.

**Approach and Results:**

First, clinical data and sera from CHD patients were collected. Then, one protein microarray containing 37 proteins that represent candidate autoantigens was developed. The arrays were used to profile autoantibodies in randomly selected sera from 35 samples (20 CHD patients, and 15 healthy controls). After that, microarray data were analyzed and autoantibodies for CHD were screened out. Then, ELISA detection was conducted to validate the differentiable autoantibodies using larger numbers of serum samples (131 CHD patients, and 131 healthy controls). Finally, the associations of antibodies with CHD risk indicator parameters were assessed. Inter-group comparison by microarray indicated that three CHD novel autoantibodies, including glucose-6-phosphate isomerase (G6PI), alpha-tropomyosin (TPM1), and heterogeneous nuclear ribonucleoprotein D-like (HnRNPDL), were significantly (*P* < 0.05) increased when compared with the healthy controls. Moreover, a significant increase of IgG autoantibodies for these three autoantigens was confirmed in CHD patients by ELISA (*P* < 0.0001). The correction analysis revealed a negative correlation of anti-TPM1 antibody levels and total cholesterol (*P* = 0.0034), and low-density lipoprotein cholesterol (*P* = 0.0086), respectively.

**Conclusion:**

G6PI, TPM1, and HnRNPDL were CHD natural autoantigens, and serum anti-TPM1 antibody could be used as a potential marker to predict the risk for CHD patients.

## Introduction

Coronary heart disease, also known as atherosclerotic vascular disease, is a common cardiovascular disease that is associated with an increased risk of mortality and morbidity (White and Chew, 2008). Globally, CHD affected 110 million people and caused about one 10th of total deaths in 2015 (World Health Organization [WHO], 2014, May). The presence of autoantibodies is a hallmark of autoimmune diseases (Fritzler, 2008). The signature autoantibodies of most autoimmune diseases have now been identified. Autoantibodies that are directed against specific proteins can reveal the stages of disease, and predict disease progression (Leeansyah et al., 2013).

Autoimmune activation, a fundamental mechanism of CHD, has been widely confirmed (Bjorkbacka et al., 2016). Current studies have shown that there are two types of autoantibodies for CHD. One type are pathological autoantibodies, and their elevation indicates a high-risk prognosis for CHD patients. The other types are protective autoantibodies from the immune system to the body. These natural autoantibodies are often produced after myocardial infarction, cardiac transplantation and pathogenic microorganism infection (Dunn et al., 1992). Although the total number of CHD autoantibodies is increasing, such as anti-Apolipoprotein B-100 antibody, anti-HSP60/65/70 a few antibodies were found in these years (Ghayour-Mobarhan et al., 2007; Zhang et al., 2010; Bjorkbacka et al., 2016). However, specific CHD antibodies have not yet been discovered (Bjorkbacka et al., 2016; Ma et al., 2017). Many circulating protein markers have been incorporated into clinical practice and have been shown to have value as CHD risk factors, but few antibody markers have been applied in clinical application as a result of a lack of basic experimental support (Keller et al., 2009). Thus, further data are still required to identify novel autoantibodies and examine their effects on CHD (Leu et al., 2017).

Conventional methods such as ELISA that are used to evaluate CHD autoantibodies are inefficient, and systematic screening is impossible (Miyashita et al., 2018). Functional protein microarrays have been designed to perform high-throughput target screening in one experiment (Song et al., 2010). It has been widely used for autoantibody detection and identification as autoimmune disease biomarkers in many biological fluids (Lueking et al., 2005; Kalbas et al., 2006). Overall, the aim of this study was to identify novel autoantibodies and measure the relationship between their titers with the prognosis of CHD autoantigen microarrays generated in our lab.

## Materials and Methods

### Serum Samples

All human serum samples were collected at Beijing Hangxing Hospital. This study was approved by the Ethics Committee at the Institute of Basic Research in Clinical Medicine, China Academy of Chinese Medical Sciences, and was conducted according to the standards of the Declaration of Helsinki. Written informed consent was obtained from participants. Patients presenting with unstable angina, non-ST-segment elevation MI, or ST-segment elevation MI, were excluded from the evaluation. Patients with severe diseases other than stable CHD were also excluded. In summary, patients with stable CHD, defined as prior MI, prior coronary revascularization, or multivessel CHD confirmed by coronary angiography, were eligible. In addition, patients had to meet at least one of the following cardiovascular risk criteria: aged ≥ 60 years, diabetes mellitus requiring pharmacotherapy, high-density lipoprotein cholesterol level < 1.03 mmol/L, current or previous smoker (defined a ≥ 5 cigarettes per day on average), significant renal dysfunction (estimated glomerular filtration rate ≥ 30 and < 60 mL/min per 1.73 m^2^, or urine albumin/creatinine ratio ≥ 30 mg albumin/g creatinine), or polyvascular disease (CHD and cerebrovascular disease or CHD and peripheral arterial disease). In total, 131 individuals with stable CHD were included. All patients denied that they had a typical history of rheumatism. In addition, serum samples were collected from 131 healthy controls, which was a convenience sample of patients who reported good to excellent health and had no diagnoses or treatment of atherosclerotic disease, hypertension, hyperlipidemia, or diabetes. Serum samples were stored in central repositories at −80°C until biochemical analysis was performed.

### Expression and Purification of Recombinant Proteins

To express the proteins, the expression plasmids were transformed into *E. coli* BL21 competent cells, respectively. The strains containing the expression plasmids were inoculated in germfree Luria-Bertani (LB) solid medium containing kanamycin 30 μg/mL. Petri dishes were left overnight upside-down at 37°C. The recombinant colonies were transformed into LB liquid medium with kanamycin 30 μg/mL and shaken for 12–14 h at 37°C. The cultures were diluted 1/100 and grown in the same medium to OD600 of 0.4–0.6, then isopropyl-D-thiogalactopyranoside (IPTG) was added to a final concentration of 1.0 mmol/L. After 5 h growth at 37°C, cells were harvested by centrifugation at 4°C and 10,000 × rpm for 20 min. Centrifuged cells were resuspended in 20 mL equilibration buffer (PBS with 8M urea, pH 7.4), disrupted by sonication (Scientz, Ningpo, China) for 1 h on ice, and the homogenate was centrifuged at 4°C and 8,000 × *g* for 20 min. Then, the supernatants of proteins were collected and purified. Recombinant proteins were purified using Ni-NTA resin (Qiagen, Hilden, Germany). The eluted proteins were stored at 4°C for use within 1 week, or at −80°C for use after a longer time. The purified proteins were analyzed on a 10% SDS-PAGE gel, and the resulting protein bands were stained with Coomassie brilliant blue R250. Purified recombinant protein was confirmed by proteomics analyzer AB 4700 mass spectrometry (Applied Biosystems, Foster City, CA, United States).

### Production of Protein Microarrays

Protein expression and purification were conducted as previously described. Printed autoantigens included 10 expressed proteins and 27 purchased antigen proteins (including classic specific in collagen diseases/rheumatic diseases antigens: SSB, dsDNA, Rib-P-1, SmD2, Rib-P-2, nucleosome antigen, nRNP, Jo-1, Sm, CENP-B, SSA, candidate CHD antigens: aldehyde dehydrogenase 1, fibrinogen, VEGF165, HSP70, Hsp60, prohibitin, vimentin, myeloperoxidase, osteopontin, myoglobin, fatty acid-binding protein, proBNP, S100B, Lp-PLA2, von Willebrand factor, brain-derived neurotrophic factor) (SinoBiological, Beijing, China), (Diarect AG, Freiburg, Germany), (Arotec, São Paulo, Brazil). These antigens are classic autoantigens and autoantigens associated with vascular lesions, and are associated with inflammation, thrombosis, and vascular lesions, and may be associated with the occurrence and prognosis of CHD. These proteins represented candidate autoantigens in CHD. Proteins were diluted to 1 mg/ml in PBS arrayed in 384-well titer dishes for printing. Purified human proteins and control proteins were spotted in duplicate onto polymer-Slide H-OP (CapitalBio, Beijing, China) at high density using a Personal Arrayer (CapitalBio, Beijing, China) (Schena et al., 1995). Every autoantigen had an average diameter of 200 μm which was printed twice. Each protein microarray included several control spots such as human IgG. Printing was performed in a cabinet at 25°C and 55% humidity. These conditions were constantly monitored by a thermohygrometer. The printed human protein chips were kept horizontal at room temperature (RT) for 1 h before storage at 4°C. Protein sequences were derived from the publicly available database UniProt.

### Serum Profiling on Protein Microarrays

Arrays were circumscribed with hydrophobic fences and blocked with PBS containing 5% fetal calf serum for 1 h at RT. The protein microarrays were incubated with a random selection of 20 CHD patients and 15 healthy controls in a total of 262 samples. Then, these microarrays were probed with 1:20 dilution of CHD patient or healthy control serum for 1 h under the same condition, followed by washing three times in PBST and incubation with a 1:400 dilution of Cy3-labeled anti-human IgG (Bioss, Beijing, China) secondary antibody for 1 h at RT in the dark. The microarray was blown dry with compressed air and scanned with a microarray scanner (CapitalBio, Beijing, China). The binding signals were acquired and analyzed using a microarray reader (CapitalBio, Beijing, China).

### Enzyme Linked Immunosorbent Assay

Validation of CHD novel autoantigen was performed with ELISA. The 96-well microtiter plates were coated with 100 μL candidate proteins (500 ng/mL, dissolved in 0.05M carbonic buffer) overnight at 4°C. After removing the liquid phase, each well was blocked with 200 μL 5% fetal calf serum (diluted in PBS) for 2 h at 37°C. Then the liquid phase was removed again and the sera of CHD patients and healthy controls (1:20, diluted in PBS) were used to incubate the 96-well microtiter plates for 1 h at 37°C, followed by washing three times in PBST and incubation with 100 μL 1:10,000 dilution of goat anti-human IgG-HRP (Bioss, Beijing, China) for 45 min. Each was washed with 0.1% PBST five times to elute the secondary autoantibodies. After that, 50 μL tetramethylbenzidine (TMB) A and 50 μL TMB B was added to each well. After keeping the plates in a dark, room-temperature location for 15 min, the reaction was stopped by adding 50 mL stop buffer (2M, H_2_SO_4_). Finally, the OD value of each well was measured with an ELISA reader (BioTek, Winooski, VT, United States) at 450 nm.

### Statistical Analysis

Binding signals were acquired using a microarray scanner, and intensity values (signal intensity – background intensity) were normalized using the mean of the positive point signals. *t*-Test statistics, Spearman’s correlation coefficients, Fisher’s exact text and cox proportional hazards regression were analyzed using SAS version 9.4 (SAS Institute, Inc., Cary, NC, United States). *P-*values of less than 0.05 were considered significant. The threshold for defining a positive result was a value higher than that of the healthy controls (mean + 2 SD).

## Results

### Characteristics of the Study Groups

The study included 131 stable CHD patients (68.0 ± 7.0 years) and 131 healthy controls (66.5 ± 8.0 years), that consisted of 66 males and 65 females, respectively. There was no statistical difference (*P* = 0.029) in age data between the two groups. The protein microarrays were incubated with a random selection of 20 patients and 15 healthy controls in the current serum samples. Table 1 shows the baseline characteristics of patients with stable CHD for protein microarrays and ELISA, with data on sex, age, body mass index (BMI), waist circumference, systolic blood pressure, diastolic blood pressure, hypertension, TC, HDL, LDL, triglycerides (TG), glucose, and tobacco. The mean age of the men and women was 66.53 and 69.48 years, respectively. The mean and median values for BMI, waist circumference, TC, HDL, LDL, TG, and glucose were within normal limits. In total 52% of CHD patients had hypertension diagnosed, and 92.31% received treatment. The mean and median values for systolic and diastolic blood pressure were not confirmed as being within normal limits. Variables were expressed as the mean (SD) when they were normally distributed and as the median and minimum–maximum range when they were not. No gender differences were found in the data. Concurrently, 131 age-gender matched healthy controls were also enrolled in this study to serve as a blank control group.

**TABLE 1:** Baseline characteristics for protein microarrays and ELISA cohorts.

Variable	Protein microarrays	ELISA
Number	20	131
Male, *n* (%)	10 (50)	66 (50.38)
Age (year)	64(47–75)	70(47–78)
BMI	26.33 (4.47)	24.87 (3.49)
Waist circumference (cm)	93.35 (14.38)	87(66.5–123)
Systolic blood pressure (mmHg)	143 (16.45)	143.48 (16.22)
Diastolic blood pressure (mmHg)	82.15 (11.38)	80.63 (9.88)
Hypertension, *n* (%)	13 (65)	52 (39.69)
On hypertensive treatment, *n* (%)	12 (60)	48 (36.64)
Total cholesterol (mmol/L)	4.92 (1.04)	4.92 (0.93)
HDL cholesterol (mmol/L)	1.22 (0.24)	1.26 (0.28)
LDL cholesterol (mmol/l)	2.90 (0.80)	2.86 (0.70)
Triglycerides (mmol/L)	1.61(0.81–6.86)	1.45(0.42–6.86)
Glucose (mmol/L)	5.84 (0.68)	5.7(4.5–7.9)

*Values in the normal distribution are shown as the mean (SD). Values in the non-normal distribution are shown as the median (minimum–maximum). BMI, body mass index; HDL cholesterol, high density lipoprotein cholesterol; LDL cholesterol, low density lipoprotein cholesterol.*

### Expression and Purification of Recombinant Proteins for Protein Microarrays

**FIGURE 1:**
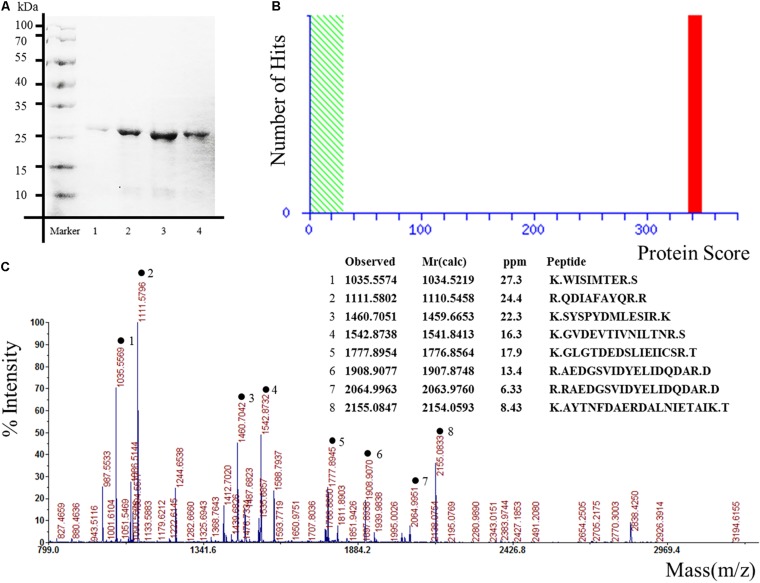
Expression, purification, and identification of recombinant proteins for protein microarrays. The recombinant proteins were expressed from the bacterial clones and purified, in parallel, under denaturing conditions. Annexin A2 was represented. **(A)** Result of scanning on SDS-PAGE gels. Lane 1–4 represented different elution times. **(B)** Ions score is –10^∗^Log (P), where *P* is the probability that the observed match is a random event. Individual ions scores > 29 indicate identity or extensive homology (*P* < 0.05). Protein scores are derived from ions scores as a non-probabilistic basis for ranking protein hits. **(C)** Proteins mass spectrometry analysis.

The pET expression system is one of the most widely used for cloning and *in vivo* expression of recombinant proteins in *E. coli* (Liu and Naismith, 2009). In order to obtain the candidate proteins, the expression plasmids pET 28 a-Hsp27, pET 28 a-Glucose-6-phosphate, pET 28 a-alpha-tropomyosin, pET 28 a-Annexin-A2, pET 28 a-JKTBP, pET 28 a-HnRNP-A2B1, pET 28 a-HnRNP-A1, pET 28 a-Moesin, and pET 28 a-Keratin 8 were transformed into *E. coli* BL21(DE3) competent cells. The results demonstrated that the proteins were purified successfully as the specific bands corresponding to the expected molecular weights of the proteins were detected by SDS-PAGE and Annexin A2 as a representative protein was displayed (Figure 1A). The proteins obtained by Ni-NTA resin were confirmed by mass spectrometry. The target proteins were identified by spectrometry analysis. The results showed that the peptide matching degree score was high, which indicated that the putative protein has a highly homologous identity (*P* < 0.05) (Figure 1B). Protein mass spectrometry is shown in Figure 1C.

### Quality Control of the Protein Microarrays

**FIGURE 2:**
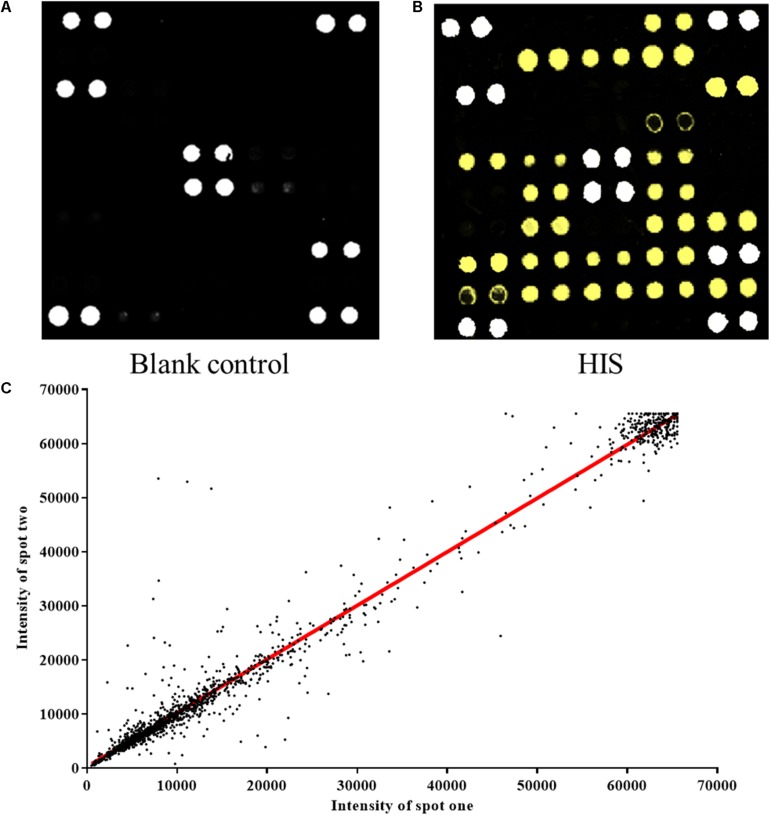
Construction of protein microarrays. **(A)** The blank control of protein microarray. BSA-Cy3 and recombinant human proteins were printed in duplicate on poly-L-lysine coated microscope slides. The BSA-Cy3 is represented in white (saturated intensity) to orient. **(B)** The protein microarray probed with anti-HIS monoclonal antibody. To monitor the quality and relative quantity of the printed proteins on glass slides, the human protein microarrays were probed with anti-HIS antibody, followed by Cy3-labeled secondary antibody to visualize the signals. The proteins positively detected by the anti-HIS antibody are represented in yellow (saturated intensity). **(C)** Correlation of spot intensities of all the duplicate pairs after experiment. The signal intensities of duplicate spots (Spot 1 versus its corresponding Spot 2) were plotted against each other. The resulting correlation coefficient was 0.9782, indicating high reproducibility of the protein spotting.

The protein microarray contained 37 proteins which represent candidate autoantigens in CHD. The printed autoantigens included 10 expressed proteins from the lab and 27 CHD related proteins that were purchased commercially. Each protein was printed repeatedly in order to ensure the quality of protein microarrays. Positive coordinate points and all other spots signal intensity in blank protein microarray were described (Figure 2A). The assay indicated that HIS-labeled proteins were detectable with anti-HIS signals, which shows that these proteins work well in the chip system (Figure 2B). All duplicate pairs after done sera experiments shows that there was a high correlation coefficient between the signal intensities of duplicate spots (Figure 2C).

### Identification of CHD-Associated Autoantigens Using Protein Microarrays

**FIGURE 3:**
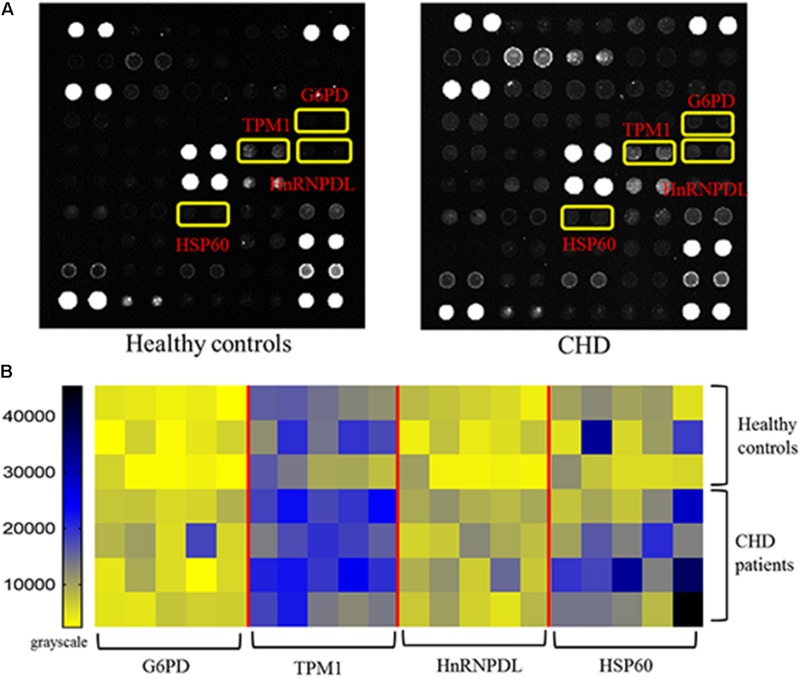
Results of protein microarrays. **(A)** Representative results of protein microarrays. The yellow boxes indicate positive candidate autoantigens between healthy controls and coronary heart disease patients, there was significant difference between two groups (*P* < 0.05). **(B)** Autoantibody reactivity was determined by 15 healthy control and 20 CHD patient serum samples. Pairwise significance analysis of microarrays was performed to identify antigen features having statistically significant.

The protein microarrays were incubated with a random selection of 20 CHD patients (Table 1) and 15 healthy controls in serum samples. Positive reactivity was detected in recombinant G6PI, HnRNPDL, TPM1 and HSP60 sera from 9, 5, 5, and 4 of 20 CHD patients, respectively. Representative results of protein microarrays are showed in Figure 3A. The yellow boxes indicate positive candidate autoantigens between healthy controls and coronary heart disease patients, there was significant difference between two groups (*P* < 0.05). The four autoantigens levels had a significant difference (*P* < 0.05) between the two groups; details of these differences are expressed in Figure 3B. For the CHD autoantigens reported in the literature, HSP60 results were similar to those previously reported (Zhang et al., 2008; Fust et al., 2012). This further illustrates the reliability of the chip method.

### Validation of Candidate Autoantibodies by ELISA

**FIGURE 4:**
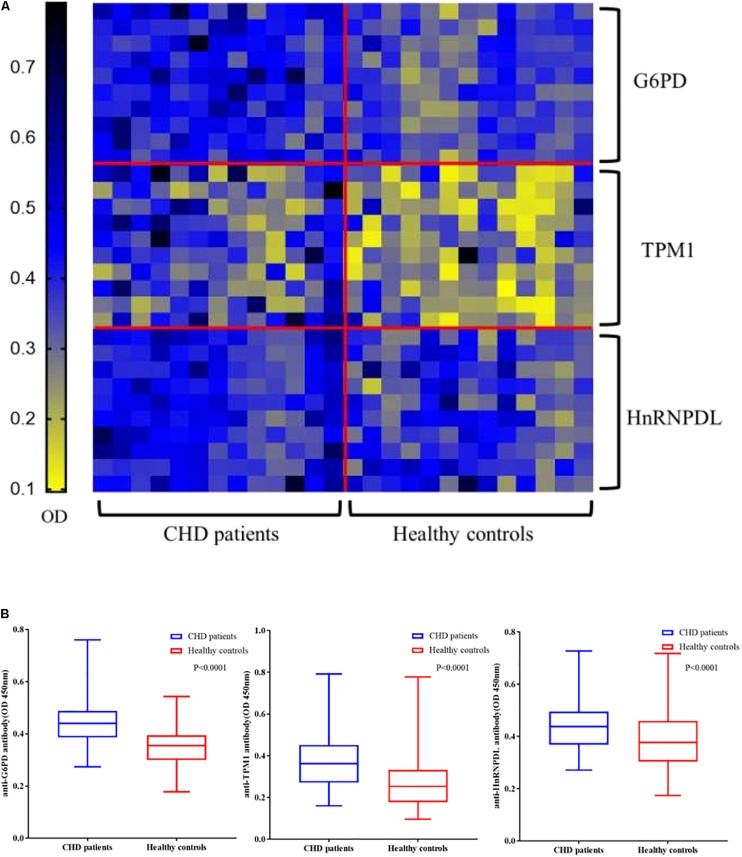
Validation of candidate autoantibodies between CHD patients and controls by ELISA. **(A)** Autoantibody reactivity was determined by 131 CHD patients and 131 healthy controls serum samples by ELISA. The OD of each of the three autoantigens (G6PD, TPM1, HnRNPDL) reacting with the serum samples in each case are displayed. **(B)** Box plots of the ELISA OD for three CHD autoantigens in various serum groups. The rectangles define the interquartile range (IQR). The bar within the rectangle indicates the median value. The bars above and below the rectangles define the 1.5I QR outlier ranges (*P* < 0.0001). OD, optical density.

Enzyme linked immunosorbent assay of three novel antigens was carried out with the sera of 131 CHD patients and 131 healthy controls to confirm the results of the microarrays. All ELISA results are shown in a heat map (Figure 4A). In large-scale samples, positive reactivity was detected in recombinant G6PI, HnRNPDL, TPM1 and HSP60 sera from 36, 22, 17 of 131 CHD patients, respectively. The reactivity of CHD serum IgG against these three autoantigens was significantly higher than healthy controls (*P* < 0.0001) in box plots (Figure 4B).

### Correlation of Autoantibodies Levels Against TC and LDL in CHD Patients

**FIGURE 5:**
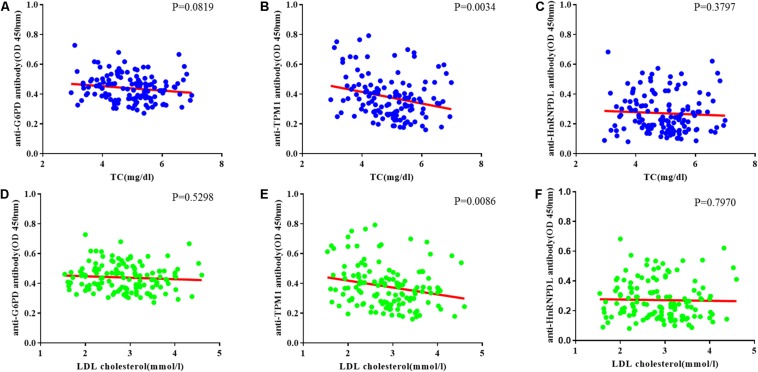
Coronary heart disease risk factor correlation analysis. **(A–C)** Relationship between total cholesterol (TC) and candidate antibodies (G6PD, TPM1, HnRNPDL) OD. There was significant correlation in anti-TPM1 antibodies (*P* < 0.05). **(D–F)** Relationship between low density lipoprotein cholesterol (LDL cholesterol) and candidate antibodies OD. There was significant correlation in anti-TPM1 antibodies (*P* < 0.05). The result showed negative correlation between antibody concentration and CHD risk factor indicators.

The relationship between TC and candidate autoantibody levels were analyzed, and there was a significant inverse correlation in anti-TPM1 antibodies (*P* = 0.0034, *r* = −0.03883) (Figures 5A–C). The same relationship was noticed for low density lipoprotein cholesterol (Ueba et al., 2009; van den Berg et al., 2018) (*P* = 0.0086, *r* = −0.04635) (Figures 5D–F).

## Discussion

The pathogenesis of CHD is associated with autoimmune activation, which can enhance atherosclerosis (Onat and Yuksel, 2013; Meurice et al., 2016). Patients suffering from an autoimmune disease, such as systemic lupus erythematosus, rheumatoid arthritis or psoriasis, display an increased CHD risk (Levy et al., 2008; Vizzardi et al., 2010; Lopez-Pedrera et al., 2012). For patients without autoimmune diseases, but established CHD, levels of natural autoantibodies against various endogenous epitopes, such as modified LDL, heat shock proteins have been shown to independently predict CHD outcome (Roux-Lombard et al., 2013; Vuilleumier et al., 2014). Also, *in vivo* and *in vitro* evidence has demonstrated that some natural autoantibodies could directly influence atherogenesis and atherosclerotic plaque vulnerability, mostly by activating innate immune receptors, thereby supporting a causal role of humoral autoimmunity in atherosclerosis (Carbone et al., 2013).

In this study, three novel autoantigens were found for CHD by analyzing microarray data; these included G6PI, HnRNPDL and TPM1. Some experimental studies confirm the hypothesis of a significant protective effect against CHD resulting from the genetic condition of G6PI deficiency (Meloni et al., 2008). G6PI also is an autoantigen widely present in rheumatoid arthritis. A high concentration of G6PI was observed on the synovial lining surface and the small artery endothelial cell surface. Whether the same mechanism exists in CHD is worthy of further study (van Boekel et al., 2001). However, this is enough to show that CHD has the property of autoimmunity. HnRNPL proteins are related to a family of heterogeneous nuclear ribonucleoproteins that function in mRNA biogenesis and mRNA metabolism (Kawamura et al., 2002). The human DNA and RNA binding protein HnRNPL is a new member of hnRNPs that are involved in mRNA biogenesis (Akagi et al., 2000). The HnRNPs family are autoantigens of many autoimmune diseases. Its release may be related to endothelial cell damage. At the same time, its ectopic expression in the cell membrane may also participate in the early mechanism of autoimmunity (Han et al., 2010).

Tropomyosin belongs to a family of actin-binding proteins that are central to the control of calcium-regulated striated muscle contraction (Jagatheesan et al., 2003). The three primary tropomyosin isoforms, α-TM, β-TM and γ-TM, are alternatively spliced products of the TPM1, TPM2, and TPM3 genes, respectively. Also, they are highly homologous but exhibit distinct physiological properties (Jagatheesan et al., 2010). Alpha-tropomyosin is a coiled-coil protein that associates with actin filaments as a homodimer and plays a central role in the calcium dependent regulation of vertebrate striated muscle contraction (Janco et al., 2012). It is a major antigen in allergic responses to invertebrates such as crustaceans, arachnids, insects, and mollusks (Reese et al., 1999). Autoimmune responses to alpha-tropomyosin have been previously reported in the sera of patients with ulcerative colitis, colonic intraepithelial lymphocytes and Behçet’s syndrome (Biancone et al., 1998; Geng et al., 1998; Mor et al., 2002).

A negative correlation of anti-TPM1 antibody concentrations and TC and LDL was found in our study. TPM1 is considered to play an important part in the regulation of the smooth muscle contraction process (Perry, 2001). The titers of these antibodies are dynamic and tend to balance with the gradual recovery of homeostasis in the body. A negative correlation between antibody titers and risk factors has been found in several studies. Low levels of anti-PC predict the development of stroke and myocardial infarction, and represent a new model from chronic disease for the body to fight against oxidized and/or inflammatory phospholipids (Frostegard, 2010). HSP-70 antibodies have also been found to have the same trend in CHD patients. The negative correlation between these natural antibodies and CHD is clearly not coincidental. We speculate that the two antibodies (G6PI and HnRNPDL) found in this study belong to pathological autoantibodies, which reflect the autoimmune properties of CHD and may damage endothelial cells and cardiac myocytes. But anti-TPM1 belongs to the regulatory natural autoantibodies in CHD. Low abundance of this antibody may indicate a decline of the body’s ability to restore homeostasis.

Current techniques to perform large-scale multiplex characterization of autoantibody responses are extremely limited. ELISA, Western-blot analysis and radio immune assays are time-consuming and tedious processes. The conception and initial development of “multianalyte microspot immunoassays” was initially proposed by Ekins (1989). MacBeath and Schreiber described spotted protein arrays as a tool to detect protein and small-molecule interactions (MacBeath and Schreiber, 2000). By enriching potential autoantigens and fabricating chips, the efficient screening of autoantigens of CHD was realized. We constructed and applied protein microarrays to perform simple, low-sample volume, fluorescence-based assays. By taking advantage of existing microarray-based data, we were able to readily identify four potential autoantigens, and then further confirm three of these proteins as newly identified CHD-specific autoantigens. Although the present study provided interesting findings about the association of novel autoantibody concentrations and the risk indicators of CHD, several potential limitations should be acknowledged. First, this pilot study and hypothesis-generating manuscript is limited by the group sample size. Second, the number of candidate autoantigens is relatively small. Finally, although the relationship between autoantibodies concentration and CHD risk indicators parameters were analyzed, more experiments are needed to understand to the mechanism of this phenomenon.

## Conclusion

We identified three novel autoantigens (G6PI, TPM1, HnRNPDL) for stable CHD and to verify the validity of HSP60. The correction analysis revealed a negative correlation of anti-TPM1 antibody levels and TC, and LDL, respectively.

## Significance

Immune responses play a key role in coronary heart disease development. Previous studies have indicated inverse associations between autoantibodies recognizing oxidized low-density lipoprotein epitopes and cardiovascular disease. The strength of the present study is the discovery of novel antibodies that allow for a unique evaluation of anti-TPM1 autoantibodies to predict risks of future cardiovascular events. Analysis of the autoantibody levels may offer an opportunity to identify individuals who are in need of immune-modulatory therapy.

## Data Availability Statement

The raw data supporting the conclusions of this article will be made available by the authors, without undue reservation, to any qualified researcher.

## Ethics Statement

This study was approved by the Ethics Committee at the Institute of Basic Research in Clinical Medicine, China Academy of Chinese Medical Sciences, and was conducted according to the standards of the Declaration of Helsinki. Written informed consent was obtained from participants.

## Author Contributions

CL, PC, AL, JY, and YZ conceived the project. PC, YZ, and CL designed the experiments. YZ, PC, and HZ carried out the research. YZ conducted the data analysis and wrote the manuscript. YZ, HZ, BL, LL, LZ, MB, XJ, XH, JY, PC, CL, and AL have read and approved the final manuscript.

## Conflict of Interest

The authors declare that the research was conducted in the absence of any commercial or financial relationships that could be construed as a potential conflict of interest.

## Abbreviations

CHD: coronary heart disease
ELISA: enzyme linked immunosorbent assay
G6PI: glucose-6-phosphate dehydrogenase
HnRNPDL: heterogeneous nuclear ribonucleoprotein D-like
IgG: immunoglobulin G
LDL: low-density lipoprotein cholesterol
TC: total cholesterol
TPM1: alpha-tropomyosin.
